# Responses of the Metabolism of the Larvae of *Pocillopora damicornis* to Ocean Acidification and Warming

**DOI:** 10.1371/journal.pone.0096172

**Published:** 2014-04-25

**Authors:** Emily B. Rivest, Gretchen E. Hofmann

**Affiliations:** Department of Ecology, Evolution and Marine Biology, University of California Santa Barbara, Santa Barbara, California, United States of America; University of Connecticut, United States of America

## Abstract

Ocean acidification and warming are expected to threaten the persistence of tropical coral reef ecosystems. As coral reefs face multiple stressors, the distribution and abundance of corals will depend on the successful dispersal and settlement of coral larvae under changing environmental conditions. To explore this scenario, we used metabolic rate, at holobiont and molecular levels, as an index for assessing the physiological plasticity of *Pocillopora damicornis* larvae from this site to conditions of ocean acidity and warming. Larvae were incubated for 6 hours in seawater containing combinations of CO_2_ concentration (450 and 950 µatm) and temperature (28 and 30°C). Rates of larval oxygen consumption were higher at elevated temperatures. In contrast, high CO_2_ levels elicited depressed metabolic rates, especially for larvae released later in the spawning period. Rates of citrate synthase, a rate-limiting enzyme in aerobic metabolism, suggested a biochemical limit for increasing oxidative capacity in coral larvae in a warming, acidifying ocean. Biological responses were also compared between larvae released from adult colonies on the same day (cohorts). The metabolic physiology of *Pocillopora damicornis* larvae varied significantly by day of release. Additionally, we used environmental data collected on a reef in Moorea, French Polynesia to provide information about what adult corals and larvae may currently experience in the field. An autonomous pH sensor provided a continuous time series of pH on the natal fringing reef. In February/March, 2011, pH values averaged 8.075±0.023. Our results suggest that without adaptation or acclimatization, only a portion of naïve *Pocillopora damicornis* larvae may have suitable metabolic phenotypes for maintaining function and fitness in an end-of-the century ocean.

## Introduction

Ocean acidification (OA) and ocean warming are expected in marine ecosystems as a consequence of continued anthropogenic fossil fuel use, with significant changes already detected in the surface ocean [1]. By 2100, human-derived atmospheric pCO_2_ (A1FI SRES [2]) absorbed by the ocean will cause seawater acidity to double [3]. Simultaneously, average sea surface temperatures will rise by 1–3°C [2]. Within marine ecosystems, we are only beginning to appreciate how regional scale variation might influence biological responses to environmental change and the adaptive potential of populations [4]–[9]. In this study, we explore one element - the physiological responses of larvae of stony coral, animals whose thermal physiology limits can be exceeded by small increases in water temperature (1–2°C; [10], [11]) or can encompass a 10°C temperature range, as a result of local adaptation, seasonal acclimatization, or thermotolerance of hosted *Symbiodinium* clades [12]. To provide an assessment of the sensitivity of coral larvae to future ocean conditions, we measured how pH and temperature interact to influence the metabolic status of the larvae of a resident coral, *Pocillopora damicornis*. To provide a relevant *in situ* environmental context for our study site, we determined the natural variation of pH and temperature on the natal coral reef during the month in which these larvae developed and released.

The study of OA has been greatly enhanced by monitoring natural pH dynamics in different marine near-shore environments. Recently, the challenge of acquiring high-frequency, long, continuous environmental datasets that estimate these changing conditions for study populations of benthic species has been overcome with the advent of autonomous oceanographic sensors that record pH [13]. Deployed and tested in sites ranging from tropical to polar, these sensors have shown that calculations of global ocean pH underestimate the natural variation in seawater pH occurring between marine ecosystems [14]–[17]. With these sensors, the research community can now collect high-frequency environmental data to complement IPCC projections and to provide details of the conditions that adults and larvae experience *in situ* [9].

As our environmental data banks grow, our knowledge about biological tolerances of interacting stressors is also increasing, particularly for stressors of OA and temperature (for recent reviews, see [18]–[23]). High pCO_2_ can exacerbate the effects of elevated temperatures by narrowing thermal tolerance windows [24]–[27]. For example, thermal sensitivity of heart rates of the spider crab, *Hyas araneus*, increased as pCO_2_ rose [27]. However, responses of physiological processes of marine invertebrates to increases in pCO_2_ and temperature have been inconsistent [28]–[32]. This variation in response to OA and its interaction with temperature may be due to differential sensitivities of particular physiological processes, like calcification, as well as taxon- and species-specific differences in sensitivity to these environmental stressors [18], [21]. Combining existing knowledge of environmental variability and potential biological responses to future ocean conditions, it is becoming increasingly clear that studies, such as this one, that present changes in biological processes at the levels of species and developmental stage in the context of environmental data are valuable for understanding how future environmental change might alter populations [33].

Tropical reef corals may be particularly vulnerable to changes in their abiotic environment. Firstly, thermal stress presents a major threat to coral reef health as the oceans continue to warm [34]. Thermotolerance limits of coral can be passed by only a few degrees of ocean warming [10], [11], and in response, bleaching often occurs, where the density of endosymbiont algae within the coral decreases. OA may act synergistically with warming to lower the temperature threshold for bleaching in reef-building corals, magnifying this threat [35]. Additionally, larvae of corals, the developmental stage that contributes to connecting populations and restoring degraded reefs, may be particularly sensitive to the interaction of OA and warming, because their metabolic demands are high while they are actively swimming and locating optimal settlement sites [36].

To better understand the consequences of OA and warming for the future of coral reefs, we examined the response of the larvae of the cauliflower coral, *Pocillopora damicornis* (Linnaeus, 1758). This branching scleractinian coral is widely distributed throughout shallow-water habitats of the Indian and Pacific oceans [37], and, like other brooding coral species, produces lecithotrophic, free-swimming larvae that are released every month in time with the lunar cycle (*e.g.* [38]–[40]). The larvae contain endosymbiotic *Symbiodinium* upon release, vertically transmitted from the parent [41]. In about 15% of coral species including *P. damicornis*, *Symbiodinium* algal endosymbionts supplement the energy available to coral larvae via transfer of metabolites [41]–[43]; the fragile symbiosis may make the holobiont more sensitive to OA and rising temperature. During their pelagic duration, *P. damicornis* larvae mainly rely on their stored lipid and translocated metabolites from the *Symbiodinium* to fuel their dispersal, settlement, and metamorphosis into the first polyp [43]. Though local retention is common [44], [45], these energy sources can allow *P. damicornis* larvae to retain competency in the plankton for more than 100 days, enough time to be carried by currents throughout the tropical Pacific Ocean [46]. Negative consequences of OA and warming on the energy budgets of *P. damicornis* larvae could thus affect dispersal distance and ultimately population dynamics for this species.

The life history strategy of corals like *P. damicornis* may provide a form of defense against the negative consequences of interacting climate change stressors on the species as a whole (*e.g.* [47]–[49]). Based on their spawning date, larval cohorts differ in terms of their inherent fitness-related traits; for example, Putnam *et al.* [49] found that larval size, symbiont density, and symbiont photophysiology vary significantly between cohorts of *P. damicornis* larvae. In another brooder, *Porites astreoides*, larval cohorts differed in symbiont density and potential for autotrophy [50]. As a result, larvae with different spawning dates may have dissimilar responses to environmental stress. While the overall proportion of fit offspring is reduced consequently, this strategy for spreading risk may increase the likelihood that some offspring have phenotypes better suited for future ocean conditions.

Although the response of coral larvae to elevated pCO_2_ and temperature is difficult to predict, in ectothermic animals, environmental stressors commonly elicit metabolic depression – a regulated reduction in metabolism in response to stress-related cues [51], [52]. Strategically, suppression of metabolism may be an effective adaptive strategy in the short-term because it prevents mortality by increasing tolerance [53]. However, when extended over long periods, low metabolic rates will likely impair growth and reproduction, decreasing fitness of the species [52], [54].

For corals and their early life history stages, metabolic depression may be a likely response to rising ocean acidity and temperature [7], [55]. Respiration rates of many life history stages increase with temperature [50], [56], [57], but narrowing of thermal tolerance windows under OA [24]–[27] may cause metabolism to decline at more conservative thermal extremes. If so, conditions of high CO_2_ and high temperature may delay or prevent larval growth and metamorphosis, increasing time in the plankton while decreasing recruitment, post-settlement success, and fitness. While elevated temperature is known to shorten the pelagic larval duration of coral larvae (*e.g.* [50], [58]), metabolic depression induced by the interacting stressors may prevent larvae from accomplishing this energetically-costly transformation. Larvae of some broadcast-spawning and brooding corals experience metabolic suppression at high pCO_2_ [59], [60] while other species are more tolerant [61]. Recent studies on larvae from a Taiwan population of *P. damicornis* have found variable responses of metabolism to elevated temperatures and a lack of response to elevated pCO_2_ [48], [62], [63]. Our study builds off this solid foundation to provide a comparison using a genetically distinct population in Moorea [64], pCO_2_ levels appropriate for current environmental variability and future projections, and insightful molecular proxies. We used environmental data collected on the reef to identify extreme conditions experienced in the field and as a context for interpreting biological responses of coral larvae to future ocean scenarios.

Studies of larval metabolism can contribute to our ability to predict the future impact of ocean acidification and warming on corals through estimates of physiological plasticity, the ability of an organism to vary the rates of physiological processes in order to maintain homeostasis as environmental conditions change [65], [66]. Making use of its existing physiological repertoire to tailor its phenotype at the cellular and molecular level to a new environmental condition, the organism has the potential for acclimatization and longer-term persistence [24], [67]. In this study, we explored plasticity of physiological responses of coral larvae from a reef in French Polynesia to elevated pCO_2_ and temperature. The study was motivated by these questions: (1) what is the response of *P. damicornis* larvae to conditions of decreased pH and warming, measured via two indices of metabolism - rates of oxygen consumption and citrate synthase activity?, (2) are there differences in larval sensitivity to environmental change between cohorts that are released from adult colonies at different times?, and (3) what is the present-day exposure of *P. damicornis* to natural variability of pH and temperature on the natal reef?

## Materials and Methods

All research, including fieldwork in Moorea, French Polynesia (17.4803 S, 149.7989 W), was performed under an annual research permit issued by the French Polynesia Ministry of Research to EBR.

### Collection of Coral Larvae

Larvae were collected from adult colonies following their lunar pattern of reproduction [68]. On the new moon (March 4, 2011), eight colonies of *P. damicornis* were collected at ∼1–2 m depth from a fringing reef site. Due to the proximity of the collection site to the oceanographic instruments, the pH and temperature histories of the adult colonies were characterized for the month prior to collection during which the larvae developed. Each colony was maintained in an aquarium at University of California Berkeley Richard B. Gump South Pacific Research Station with indirect natural sunlight and a slow flow of coarsely filtered seawater. Temperatures in these aquaria averaged 28.4±0.4°C throughout the spawning period. Overnight, larvae were captured in mesh-lined cups that received the outflow of each aquarium. Daily at dawn, larvae from each colony were collected, counted, pooled, and randomly assigned to experimental treatments. Although there was daily variation in larval output between colonies, low release levels in general necessitated the use of all larvae in the daily experiments. Uneven contributions of genotypes in the experimental larval pool each day were unavoidable and preclude any distinction between genotypic responses and species-level responses to OA and temperature. Data presented here were collected from manipulative experiments conducted with larvae released on March 13 (“Day 9″), March 14 (“Day 10″), and March 15 (“Day 11″).

### Experimental Incubations

Two CO_2_ treatments were prescribed: Low-pCO_2_ (∼450 µatm CO_2_) and High-pCO_2_ (∼950 µatm CO_2_). The low treatment represents an environmental condition that released larvae may currently experience at this site (approximated by environmental data), while the high treatment represents a level of dissolved pCO_2_ that is outside the present-day pH minima of the seawater bathing the fringing reef and that is a surface ocean average expected by the year 2100 under the A1FI scenario [69]. These conditions approximate those at the reef scale and not what may be experienced and manipulated within the boundary layers of the adult corals. pCO_2_ levels were combined with two experimental temperatures, 27.8°C and 30.6°C. The control temperature (27.8°C) approximates the 5-year average temperature at Moorea Coral Reef Long Term Ecological Research (MCR LTER) monitoring site close to the collection site for adult *P. damicornis* as well as a verified environmental condition during the month preceding and including the release of larvae used in this experiment. The elevated temperature represents the average surface ocean temperature by year 2100 as predicted by global temperature projections [70].

Treatments were created as described in Edmunds *et al.* [71] in a MCR LTER facility, with one aquarium for each treatment combination of pCO_2_ and temperature. Tank replication was not possible due to unexpected equipment failure. The closed-circuit aquaria were filled with 20 µm-filtered seawater, 16% of which was replaced daily. Gas mixtures of the two desired CO_2_ levels were created following Edmunds *et al.* [71] and then bubbled directly into experimental aquaria. Saturation of pCO_2_ in the seawater was reached before each daily experiment was performed. Individual Aqua Logic aquarium heaters and a chill loop maintained tank temperature treatments at +/−1°C. Aquaria were darkened with aluminum foil. The four treatments created by this experimental set-up are defined as low temperature-low pCO_2_ (LTLC), low temperature-high pCO_2_ (LTHC), high temperature-low pCO_2_ (HTLC), and high temperature-high pCO_2_ (HTHC).

To verify and monitor the physical parameters of the OA x temperature treatments, the chemistry of the seawater in the aquaria was analyzed daily. pH, temperature, salinity, and total alkalinity of seawater in each aquarium were measured during the incubations.

Seawater temperature was measured throughout the experimental exposures (5–6 times) using a thermocouple (T-type, Omega Digital Thermometer, Model HH81A). Seawater salinities were measured using a conductivity meter (YSI 3100). Seawater pH was measured using a spectrophotometric method with indicator dye, *m*-cresol purple (SOP 6b [72]). Total alkalinity (A_T_) was measured using an automated, open-cell potentiometric titration (SOP 3b [72]) with a Mettler-Toledo T50 titrator and a DG115-SC pH probe (Mettler-Toledo). Titrations were performed using certified acid titrant (∼0.1 M HCl, 0.6 M NaCl; A. Dickson Laboratory, Scripps Institute of Oceanography), and a non-linear least-squares approach was used to calculate A_T_ [73]. For each day of the experiment, analyzed certified reference materials from A. Dickson Laboratory were accurate within 10 µmol kg^−1^. pH at 25°C, A_T_, temperature, and salinity were used to calculate the pH and pCO_2_ of the treatments using CO2calc [74], with CO_2_ constants K1, K2 from [75] refit by [76] and pH expressed on the total scale (mol kg-SW^−1^).

### Assessment of Physiological Responses

To assess how larval metabolism responds to OA and warming, larvae were placed in 10 mL serum vials that contained seawater filtered to 0.2 µm from aquaria at all four combinations of temperature and pCO_2_. For each treatment combination, there were 6 vials containing 5 larvae each and two blank vials. Each vial was sealed with parafilm so that no air bubbles remained inside (for optimization of methodology, see File S1). To account for any change in chemistry as treatment water was filtered to 0.2 µm and used to fill the vials, treatment water was measured before and after the vials were loaded (see Table 1, rows ‘Vials’). The loaded vials were incubated for 6 hours in the dark treatment aquarium (the source for the water used to fill the vials). Due to the time needed to read the oxygen concentration in the vials post-incubation, loading of the vials for each treatment was staggered by one hour with the order randomized daily. Vials were cleaned and re-used for respirometry incubations on subsequent days.

**Table 1:** Summary of physical conditions in treatment aquaria and vials for experiments conducted on Days 9–11.

Treatment		Temperature (°C)	Salinity (ppt)	pH	A_T_(µmol kg^−1^)	pCO_2_ (µatm)
LTLC	Tank	27.5	35.33	8.018	2353±13	435.93±6.00
	Vials			7.995		464.40±5.96
HTLC	Tank	30.7±0.1	35.40	7.985	2364±7	476.62±3.60
	Vials			7.994		465.61±5.91
LTHC	Tank	28.1	35.43	7.714	2354±16	995.54±11.82
	Vials			7.726		965.35±16.05
HTHC	Tank	30.44±0.1	35.63±0.1	7.736	2383±5	952.10±9.98
	Vials			7.759		894.91±10.93

Data are presented as mean ± SE, except where SE <0.1. For all parameters, *n* = 3.

In order to measure oxygen concentration, approximately 325 µL of the seawater in each vial was injected into a glass custom-built optrode cell. One measurement of oxygen consumption was made per vial. A built-in water jacket surrounding the optrode cell was connected to a re-circulating water bath held at the same treatment temperature of the vials being analyzed. After two minutes, the oxygen concentration was read in triplicate (Microx TX3, Presens GmbH, Regensberg, Germany). Oxygen consumption over the 6-hour incubation was calculated (nmol O_2_ larva^−1^ min^−1^), and batches of 5 larvae from each respiration vial were preserved for analysis of total protein in order to account for variation of larval mass within a daily cohort. While *Symbiodinium* numbers were not accounted for, variation in endosymbiont density is unlikely to affect the biomass-standardized rate of respiration [77].

To complement oxygen consumption rates, citrate synthase (CS) activity was quantified to gauge changes in larval oxidative capacity in response to the OA x temperature treatments. Additional larvae were incubated for 6 hours simultaneously with those in the respirometry vials and then frozen at −80°C. Within each treatment, these larvae were incubated at a density of 1 larva mL^−1^ in three flow-through 50 mL Falcon tubes enhanced with 100 µm-mesh windows.

CS activity in homogenates of larvae of *P. damicornis* was measured spectrophotometrically according to Srere [78] as modified by preliminary tests to determine optimal pH and substrate concentrations (File S2). To quantify CS activity, larvae were first homogenized on ice in 50 mM histidine pH 7.8 using a pestle followed by further physical disruption using a pipettor. Centrifugation (5 min at 13,362×g) was used to separate *Symbiodinium* cells from animal homogenate with minimal animal mitochondria in the pellet. Aliquots of the homogenates were preserved for later analysis of total protein. Triton X-100 was added to the remaining homogenate at a final concentration of 0.25% v/v. At 28.0°C ±0.1°C, the control temperature for culturing and respiration, absorbance at 412 nm of the reaction was measured with and without oxaloacetate, with final concentrations of 0.4 mM acetyl coA, 0.25 mM DTNB, and 0.5 mM oxaloacetate. Measurements of CS activity were technically replicated for each tube of larvae (*n* = 2). Rates of CS activity are expressed as µmol min^−1^ larva^−1^ and are also standardized by total protein to represent protein-specific activities (µmol min^−1^ g animal protein^−1^ [79], [80]).

Total protein values were used to normalize data for oxygen consumption and CS activity. Following sonication, total protein content of larvae was determined using a Bradford assay [79], [80].

Temperature coefficient Q_10_ values were calculated to determine if the sensitivity of larval metabolism to temperature changed under different pCO_2_ levels. Q_10_, commonly used to describe the sensitivity of reaction rates to temperature, is the factor by which the reaction rate increases following a 10-degree increase in temperature. To calculate this coefficient, the following formula was used: Q_10_ = (R_2_/R_1_)∧(10/(T_2_–T_1_)), where R is the rate of reaction at Temperature 1 or Temperature 2. Q_10_ values of biological reaction rates are commonly between 2 and 3 (*e.g.* [81]). Values below 2 indicate a decrease in temperature sensitivity while values above 3 indicate hypersensitivity of the reaction to changes in temperature.

### Statistical Analysis

All data were analyzed using R version 3.0.1 (R Core Team 2013). A one-way ANOVA in which pCO_2_ and temperature were fixed factors was used to compare physical conditions between treatments. With pCO_2_, temperature, and day of release as fixed factors, effects on larval- and protein-specific rates of oxygen consumption, CS activity, and total and animal protein levels were estimated using linear mixed-effect models (nlme package in R [82]). To account for possible similarities between larvae incubated in the same container, “tube” was considered a random factor in all statistical analyses. Model selection was performed incrementally following Burnham and Anderson [83]. At each iteration, the simpler model was chosen if the model AIC value did not increase by 2 or more and if there was not a significant difference in the model log likelihood ratio. Effects of fixed factors were compared using likelihood ratio tests conducted on selected models fit using maximum likelihood [84], [85]. When significant differences were detected among treatments, orthogonal contrasts were performed as post-hoc analyses using the multcomp package in R [86]. Tukey’s HSD was used for models without significant interactions between terms. When significant interactions were present, post-hoc analyses were performed using linear contrasts with Bonferonni corrections for multiple comparisons. In all cases, statistical assumptions of normality and homogeneity of variance were met.

### Collection of Environmental Data

pH and temperature time series were generated on a fringing reef in Moorea, French Polynesia. pH was recorded continuously from January 28 to March 19, 2011 on the fringing reef approximately 90 m from the collecting location of adult *P. damicornis* parents. An autonomous data logger based on a Honeywell Durafet pH sensor, called a SeaFET [13], was deployed at 17.4803 S, 149.7989 W. The SeaFET was deployed at 3.3 m depth and suspended approximately 0.6 m off the sandy bottom; the instrument measured pH voltage at 10-minute intervals, averaging data over 30-second periods. The sensor reference anomaly oscillated between ±0.01 and no detectable drift of the instrument occurred. Additionally, output of continuous operation of this sensor over a 6-month period has been shown to match frequent discrete samples of seawater chemistry [13]. Adjacent to the SeaFET were two Seabird thermisters (SBE 39), synced with the SeaFET to simultaneously record temperature.

Following deployment, the SeaFET electrodes were calibrated using discrete seawater samples collected *in situ*, justified based on sensor characteristics previously demonstrated [13]. On February 25, 2011, a SCUBA diver using a Niskin bottle collected a single calibration sample adjacent to the SeaFET in concurrence with its voltage reading. Temperature of the seawater *in situ* was measured using an alcohol thermometer. pH, total alkalinity (A_T_), and salinity were measured in four replicates (see below) within 1–2 hours of sample collection. Average A_T_ and salinity values were used to generate *in situ* pH, Ω_arag_, Ω_calc_, and pCO_2_ values using CO2 calc [74]. For purposes of calculation, salinity and total alkalinity were assumed constant throughout the 2-month deployment, allowing us to generate a real-time graph of pCO_2_ variation over a coral reef. These assumptions were necessary because these parameters could only be measured using discrete samples. To estimate the error introduced in the pCO_2_ calculations by our assumptions, we used discrete bottle samples to estimate changes in the carbonate chemistry, salinity, and TA at the deployment site following a rain event.

### Data Access

Environmental (accn #: knb-lter-mcr.2004) and physiological (accn #: knb-lter-mcr.2008) datasets generated by this study are publicly available in the LTER Metacat data catalog, mirrored in DataONE.

## Results

### Larval Production

**Figure 1:**
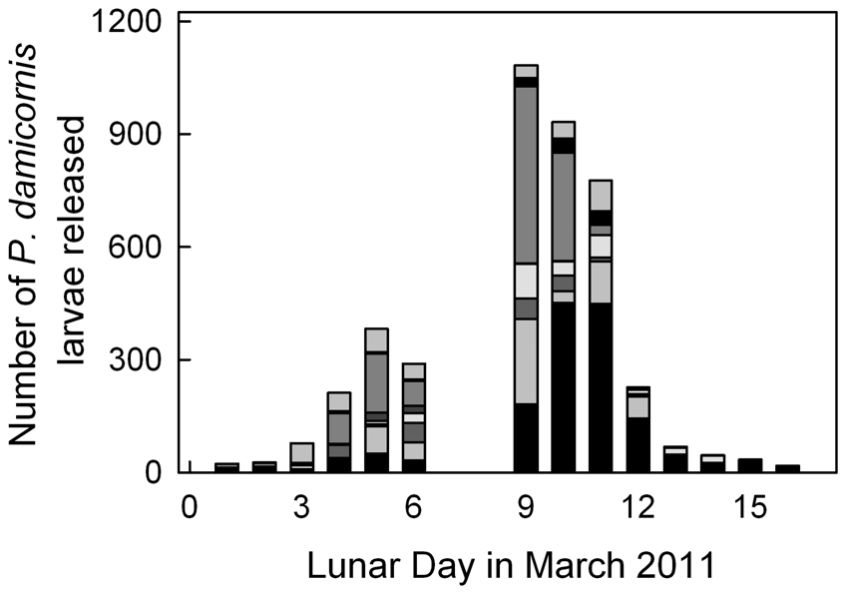
Release of *Pocillopora damicornis* larvae in March 2011. Larval release increased following the new moon and then decreased after lunar day 9. Numbers of larvae released per colony (*n* = 8 colonies) are described by bar segments of different colors.

During the period of this experiment, *P. damicornis* colonies released planula larvae for 16 days following the new moon in March 2011 with variation in the number of larvae released (Fig. 1). The peak day of larval release for this representative population was Lunar Day 9 (Fig. 1). Larval release was not counted on Lunar Days 7 and 8 due to a tsunami warning and a power outage.

### Physiological Response of Larvae to Controlled pH Variation

On several days during this pattern of larval release (Lunar Days 9–11), we tested the performance using two indicators of metabolism – oxygen consumption and CS activity, an indicator of oxidative capacity.

In order to assess the response of coral larvae to present and future pCO_2_ and temperature levels, larvae were exposed to a set of conditions in the lab where the temperature and seawater chemistry in the experimental aquaria were carefully controlled (Table 1). Experimental treatment conditions remained stable throughout the course of the experiment and grouped by treatment, despite slight differences between aquarium and vial conditions (File S3).

**Figure 2:**
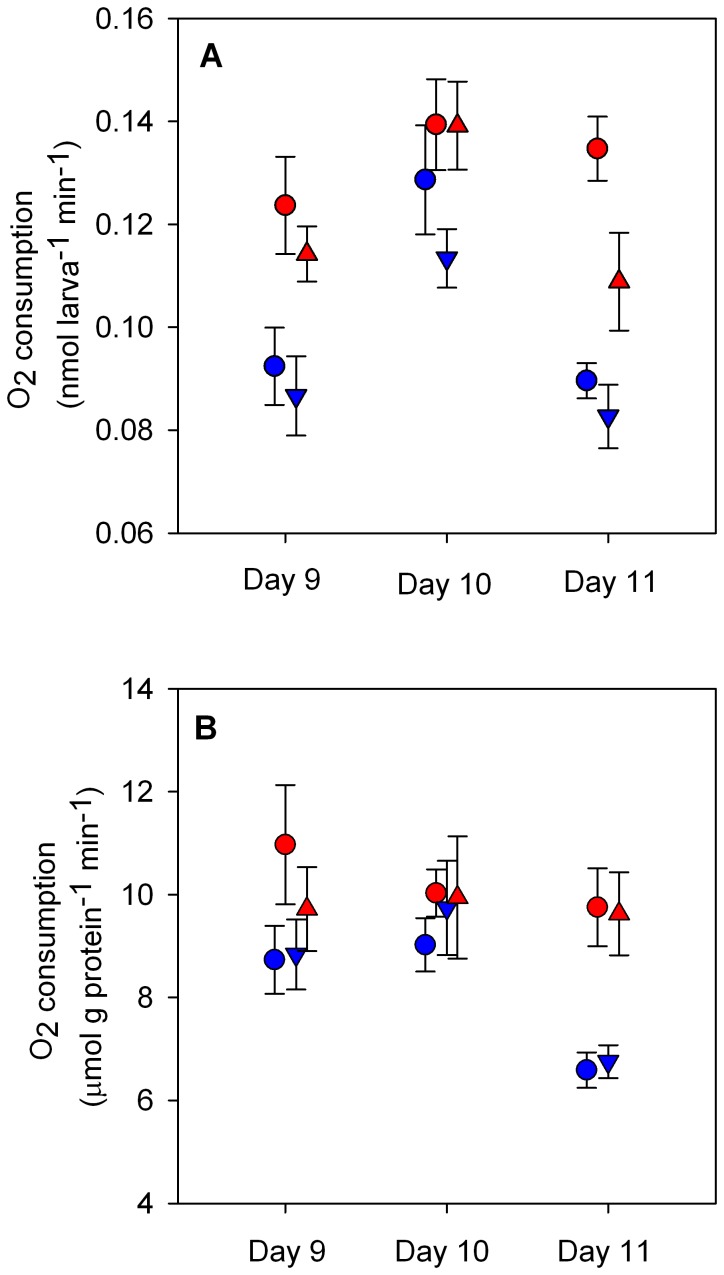
Oxygen consumption of *Pocillopora damicornis* larvae over 6-hour exposures to combinations of pCO_2_ and temperature. Mean ± SE (*n* = 6) rates of oxygen consumption standardized by number of larvae for those released on Days 9 - 11 (A) and standardized by total protein for larvae released on Days 9 - 11 (B). Larval respiration is significantly higher at 30.6°C (vs. 27.8°C), at Low-pCO_2_ (vs. High-pCO_2_) and on Day 10. Protein-specific rates are significantly higher at 30.6°C on Day 11 only. Refer to Table 2 for statistical details. Symbols are offset to improve clarity: Low-pCO_2_ at 450 µatm (circles), High-pCO_2_ at 950 µatm (triangles), 27.8°C (blue), and 30.6°C (red).

Oxygen consumption, used as an indirect measure of metabolism, showed that larvae were sensitive to projected end-of-the-century ocean chemistry. Oxygen consumption of *P. damicornis* larvae varied between 0.0826±0.006 nmol larva^−1^ min^−1^ (Day 11 LTHC) and 0.1394±0.009 nmol larva^−1^ min^−1^ (Day 10 LTHC). With no significant interactions between fixed effects, larval specific oxygen consumption varied significantly by pCO_2_, temperature, and day (Table 2). In general, rates of oxygen consumption per larva were higher at Low-pCO_2_ (vs. High-pCO_2_; Tukey’s HSD; *p* = 0.0133; Fig. 2A) and at 30.6°C (vs. 27.8°C; Tukey’s HSD; *p*<0.0001). Additionally, larvae released on Day 10 respired more quickly than larvae released on the other two days (Tukey’s HSD; *p*<0.0001 for both). Larval rates of oxygen consumption were on average 22.4% higher at 30.6°C vs. 27.8°C. The significant effect of CO_2_ across days is driven by the lower oxygen consumption rates at 30.6°C on Day 11. At 30.6°C, Day 11 larvae at High-pCO_2_ consumed oxygen 19.2% more slowly than larvae at Low-pCO_2_, compared with 7.6% on Day 9 and 0.1% on Day 10 (Fig. 2A).

**Table 2:** Analysis of oxygen consumption rates for *P. damicornis* larvae among treatments, standardized to number of larvae (nmol larva^−1^ min^−1^) and to total protein (pmol µg protein^−1^ min^−1^).

Dependent variable	Effect	X^2^	Degrees of Freedom	*p*
Larval-specific	pCO_2_	6.662	1	0.0098
oxygen consumption	T	51.863	1	<0.0001
(nmol larva^−1^ min^−1^)	Day	54.140	2	<0.0001
Protein-specific	pCO_2_	0.0879	1	0.7669
oxygen consumption	T	5.1395	1	0.0234
(pmol µg protein^−1^ min^−1^)	Day	18.2335	2	0.0001
	T x Day	5.3787	2	0.0679
Total protein	pCO_2_	4.186	1	0.0408
(µg larva^−1^)	T	2.469	1	0.1161
	Day	20.110	2	<0.0001

Comparisons were made using a three-way ANOVA with pCO_2_, temperature (T) and day of release (Day) as fixed effects. Interaction terms that were removed from the model are not shown here.

To complement calculations of metabolic rate on a per larva basis, oxygen consumption was standardized by total holobiont protein, which varied significantly by pCO_2_ and Day (Table 2, File S4). Protein-specific rates account for differences in mass between replicates and treatments whereas larval-specific metabolism allows for interpretation of physiological response in ecological units, at the level of the whole animal. When protein-specific rates of oxygen consumption were compared, elevated pCO_2_ no longer caused a significant decrease in oxygen consumption for larvae released on Days 9–11 (Table 2, Fig. 2B). Effects of T and Day remained significant (Table 2). Post-hoc analysis of the marginally significant interaction (T x Day, Table 2) revealed significant effects of temperature on Day 11 (linear contrast with Bonferroni correction; Ζ = −4.045, *p* = 0.0002), but no difference between temperatures on Days 9 and 10. The effect of temperature on protein-specific rates of oxygen consumption on Day 11 was 2-fold greater than on the other days.

**Figure 3:**
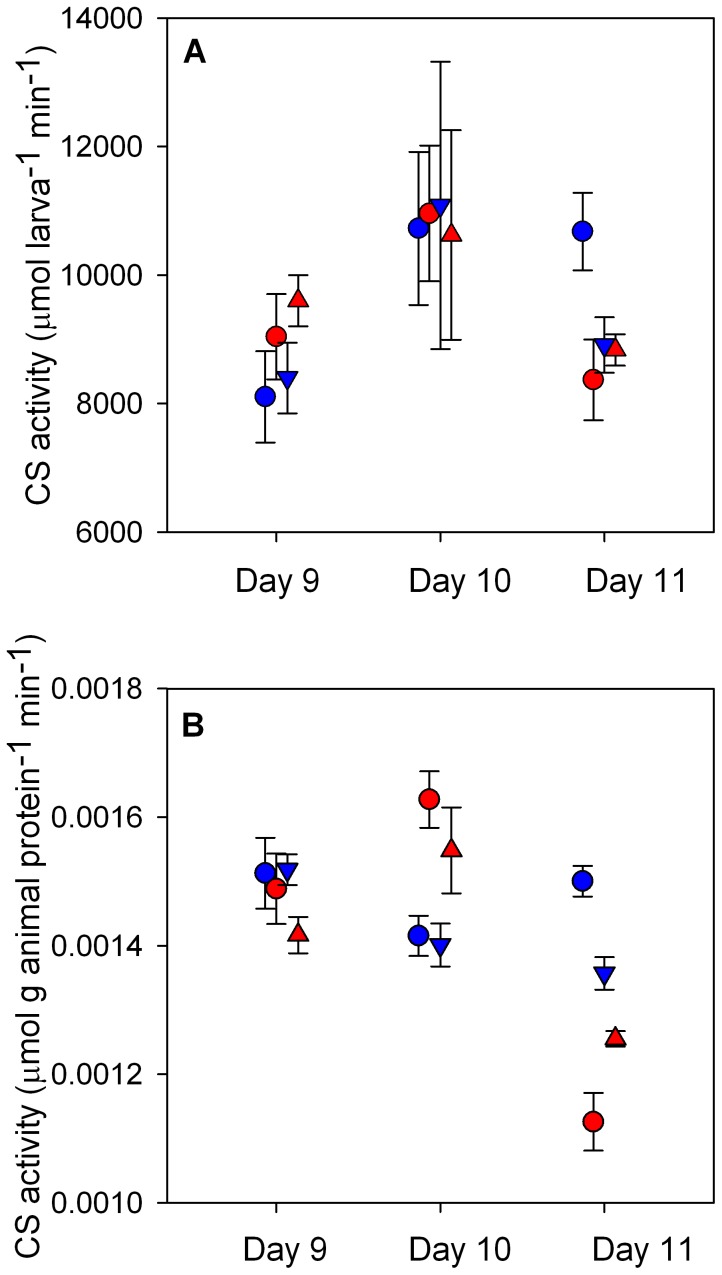
Citrate synthase activity of *Pocillopora damicornis* larvae over 6-hour exposures to pCO_2_ and temperature. Mean ± SE (*n* = 6) rates of citrate synthase (CS) activity standardized by number of larvae for those released on Days 9–11 (A) and standardized by animal protein content for larvae released on Days 9–11 (B). Refer to Table 3 for statistical details. Symbols are offset to improve clarity: Low-pCO_2_ at 450 µatm (circles), High-pCO_2_ at 950 µatm (triangles), 27.8°C (blue), and 30.6°C (red).

Citrate synthase (CS) activity was measured as a proxy for the number of intact mitochondria and to quantify the capacity of larval aerobic metabolic machinery [87]. With respect to numbers of larvae, coral animal CS activity differed by Day, marginally by T and not by pCO_2_. Despite a significant interaction between T and Day (Table 3), post-hoc analyses using linear contrasts with Bonferroni corrections showed insignificant differences between temperature groups on each day. On Day 9, elevated temperature raised CS rates slightly while on Day 11, CS activity was suppressed by elevated T and/or pCO_2_ in general (Fig. 3A). When coral animal CS activities were normalized to total protein from the animal fraction of the larval holobiont, activities ranged from 0.00136±0.0003 µmol g animal protein^−1^ min^−1^ (Day 11 HTLC, Fig. 3B) to 0.00163±0.00004 µmol g animal protein^−1^ min^−1^ (Day 10 HTLC). Protein-specific CS activities varied significantly by T x Day, and pCO_2_ x T x Day (Table 3). Determined using post-hoc analyses of linear contrasts with Bonferroni corrections, there were no significant contrasts among treatment groups on Day 9. Day 10 CS activity HTLC was significantly greater than LTLC (Ζ = 3.513, *p* = 0.0080) and LTHC (Ζ = 3.753, *p* = 0.0032). On Day 11, protein-specific CS activity for LTLC was significantly greater than for both high-temperature treatments (HTLC: Ζ = 6.207, *p*<0.0001; HTHC: Ζ = 4.063, *p* = 0.0009). Treatment groups LTHC and HTLC were also significantly different (Ζ = 03.829, *p = *0.0023). While protein-specific rates of CS activity do not yield information regarding relative amounts of CS with respect to the pool of total animal protein (see File S4), they reflect differences in activity per enzyme unit, changes in the proportion of CS within total protein, or a combination of both.

**Table 3:** Analysis of citrate synthase (CS) activity for *P. damicornis* larvae among treatments, standardized to number of larvae (µmol larva^−1^ min^−1^) and to animal protein content (µmol g animal protein^−1^ min^−1^).

Dependent variable	Effect	X^2^	Degrees of Freedom	*p*
Larval-specific	pCO_2_	0.0140	1	0.9058
CS activity	T	3.4276	1	0.0641
(µmol larva^−1^ min^−1^)	Day	21.7758	2	<0.0001
	T x Day	8.7063	2	0.0129
Protein-specific	pCO_2_	0.0082	1	0.9278
CS activity	T	0.1636	1	0.6858
(µmol g animal	Day	3.0808	2	0.2143
protein^−1^ min^−1^)	pCO_2_ x T	0.8207	1	0.3650
	pCO_2_ x Day	3.1308	2	0.2090
	T x Day	40.1282	2	<0.0001
	pCO_2_ x T x Day	8.1727	2	0.0168
Total animal protein	pCO_2_	0.1910	1	0.6621
(µg larva^−1^)	T	6.5781	1	0.0103
	Day	37.1980	2	<0.0001
	T x Day	12.0144	2	0.0025

Comparisons were made using a three-way ANOVA with pCO_2_, temperature (T) and day of release (Day) as fixed effects. Interaction terms that were removed from the model are not shown here.

We can describe how OA affects metabolism by comparing how the temperature coefficient Q_10_ values of these reactions change under different CO_2_ levels. For larval-specific O_2_ consumption, Q_10_ values ranged from 1.26 to 3.89 (Table 4). When O_2_ consumption was normalized with protein content, Q_10_ values remained under 2 except for larvae released on Day 11. Regardless of the standardization type, Q_10_ values varied considerably among lunar days. On some days, High-pCO_2_ increased the thermal sensitivities of oxygen consumption rates; on others, it depressed their temperature dependencies (Table 4). For CS activity, larval-specific Q_10_s ranged from 0.44 to 1.80 (Table 4). Protein-specific Q_10_s fell under 1.0 except for larvae released on Day 11. Again, Q_10_ values as well as the directional effect of pCO_2_ on Q_10_ varied among days (Table 4).

**Table 4:** Q_10_ values for rates of O_2_ consumption and citrate synthase activity of *P. damicornis* larvae incubated for six hours in seawater at different temperature and CO_2_ levels.

Dependent variable	Batch of Larvae	Q_10_ at Low-CO_2_	Q_10_ at High-CO_2_	Delta Q_10_
Larval-specific	Day 9	2.38	3.37	0.98
O_2_ consumption	Day 10	1.26	2.59	1.33
	Day 11	3.89	2.83	−1.06
Protein-specific	Day 9	1.98	1.52	−0.45
O_2_ consumption	Day 10	1.37	1.10	−0.26
	Day 11	3.70	3.81	0.11
Larval-specific	Day 9	1.39	1.80	0.41
CS activity	Day 10	1.07	0.82	−0.25
	Day 11	0.44	0.97	0.52
Protein-specific	Day 9	0.95	0.74	−0.21
CS activity	Day 10	1.51	1.59	0.08
	Day 11	0.38	0.75	0.36

### Natural Variability in pH and Temperature Proximal to the Natal Reef

**Figure 4:**
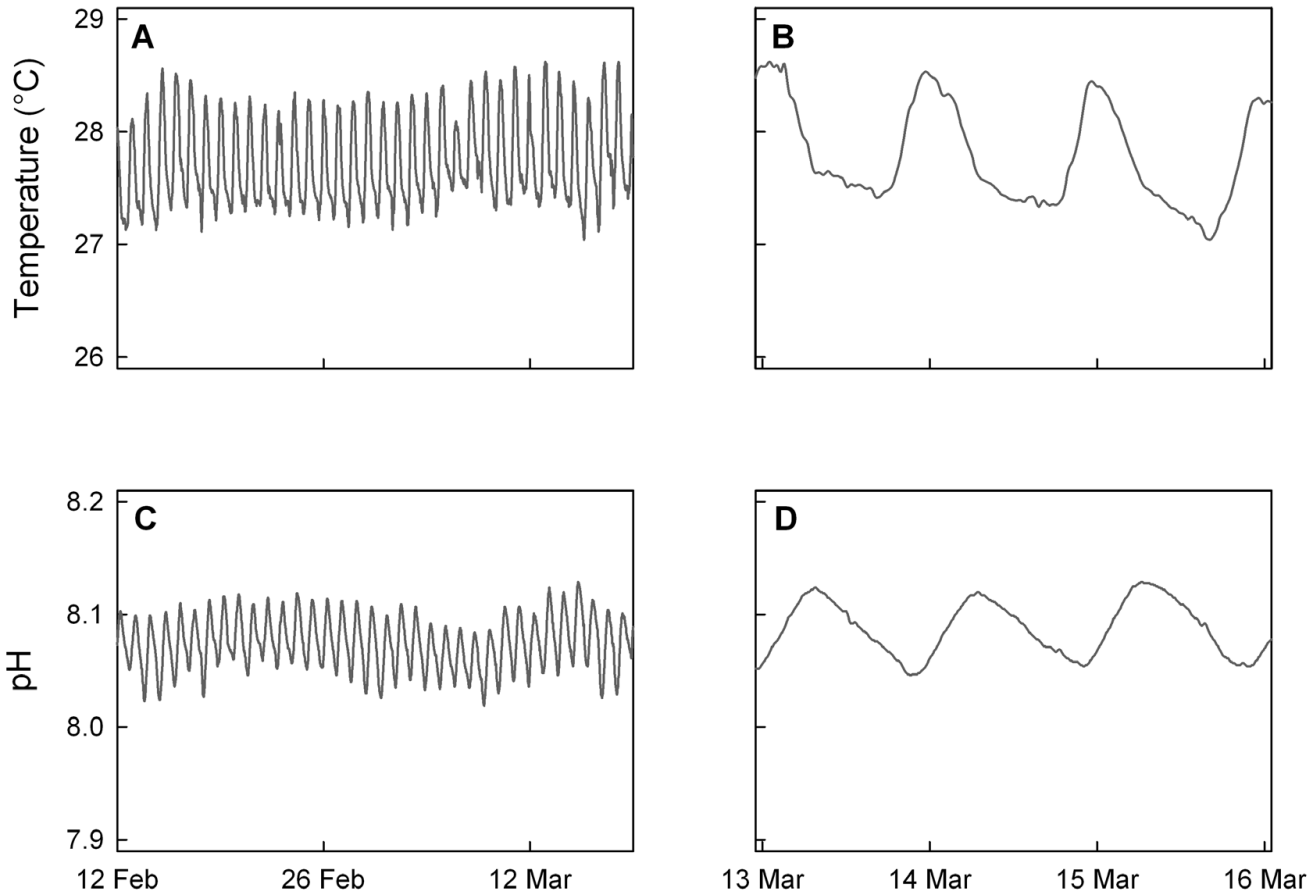
Time series of temperature and pH at a fringing reef in Moorea, French Polynesia. During the month prior to larval release, environmental temperature (A) and pH (C) oscillated on a 24-hour period. A three-day window, March 13–15, 2011, corresponds to ambient temperature (B) and pH (D) conditions adjacent to the natal reef of the larvae used in manipulative experiments on those days. Temperatures represent averages from duplicate thermisters, processed by a one-hour low-pass filter. pH data from a SeaFET sensor were processed by a one-hour low-pass filter.

Data for the present-day environmental exposures for coral larvae released on a Moorea fringing reef were measured using a SeaFET sensor near the coral collection site. Two key observations were made: (1) pH varied with a diel pattern, and (2) the delta pH (maxima minus minima) was 0.110 pH units. Throughout the 50-day deployment (data from 36 days shown here), pH and temperature fluctuated consistently through time with both parameters oscillating on a diel cycle with minima and maxima reached once every 24 hours (Fig. 4). pH values between 8.019 and 8.129 were recorded, with a mean value of 8.075 (Table 5). With regard to the nature of the diel pattern, pH maxima occurred on average around 06∶14 UTC (20∶14 local time), almost two hours after sunset, and pH minima occurred on average around 20∶59 UTC (10∶59 local time), almost five hours after sunrise. Temperature oscillated on a 24-hour period between 27.04°C and 28.62°C, averaging 27.73°C (Fig. 4A; Table 5). Daily minima and maxima occurred at approximately 14∶00 and 02∶00 UTC (04∶00 and 16∶00 local time), respectively. Fluctuations in pH lagged behind those in temperature by 4–7 hours. Temperature fluctuated according to the photoperiod with coldest temperatures occurring two hours before dawn and warmest temperatures occurring two hours before sunset.

**Table 5:** Summary of oceanographic conditions on a fringing reef in Moorea, French Polynesia from February 12– March 19, 2011 (UTC).

Summary Statistics	Temperature (°C)	pH	Ω_arag_	Ω_calc_	pCO_2_ (µatm)
*n*	25841	5169	5169	5169	5169
Mean	27.730	8.075	4.022	6.049	374.27
SD	0.376	0.0229	0.155	0.232	24.86
Range	1.578	0.110	0.698	1.049	117.89
Max	28.620	8.129	4.378	6.587	437.33
Min	27.042	8.019	3.680	5.538	319.44
25%	27.421	8.058	3.903	5.871	354.32
75%	28.048	8.093	4.147	6.238	392.44

Temperatures were recorded by duplicate thermisters. pH (in total scale) was recorded by a SeaFET. Salinity and A_T_ were measured from a discrete seawater sample collected on February 24, 2011: S = 35.1; A_T_ = 2370.16.

Despite measurable changes in seawater chemistry associated with daily fluctuations, aragonite saturation states remained adequate for coral calcification (Ω_arag_ >3.5 [88]). pCO_2_ fluctuated between 319 and 437 µatm with an average of 374 µatm (Table 5). At this site, changes in A_T_ are most likely due to freshwater influence following precipitation runoff from land. After a rain event, salinity at this site can decline up to 1 ppt, while A_T_ can fall by 50 µmol kg seawater^−1^ (E. Rivest, unpublished data). Variations in salinity and A_T_ alter the calculated pCO_2_ by approximately 5–10 µatm for the range of pH recorded at this site. Similarly, uncertainties for Ω_arag_ and Ω_calc_ are ∼0.06 and ∼0.04, respectively. We can interpret the descriptive statistics shown in Table 5 with this uncertainty in mind, but the diel oscillation in pH remains distinct.

## Discussion

To assess metabolic plasticity during early dispersal of *Pocillopora damicornis* larvae, we performed laboratory experiments whose treatment conditions were determined based on environmental extremes recorded at the collection site. These microcosm experiments tested effects of future projected ocean conditions. As indicators of performance under conditions of OA and warming, we used oxygen consumption and citrate synthase activity. Our results indicate that *P. damicornis* larvae differ in their sensitivity to environmental change with respect to the day their cohort was released from adult colonies.

### Metabolic Status under Multiple Stressors

In general, high pCO_2_ did not increase, but decreased, the demands of aerobic metabolism in *P. damicornis* larvae (Fig. 2A). Specifically, for the early release larvae on Days 9 and 10, changes in O_2_ consumption were smaller, while on Day 11, larvae exhibited more distinct signs of metabolic depression under high pCO_2_, high temperature conditions. With our small sample size of 6, the differences could be due to variation in the size of individuals in the treatments due to chance. Contrasting the results obtained for pCO_2_ levels, temperature had a more consistent effect on larval oxygen consumption (Fig. 2A). Higher respiration rates at 30.6°C were expected, as body temperature (*i.e.* ambient temperature for coral larvae) and whole-organism metabolic rate are highly correlated [89] via the kinetics of biochemical reactions. Despite the increased demand for energy that concurrent elevated temperature and pCO_2_ likely imposed, the effects of these stressors - a direct effect on molecular kinetics and an increased cost of maintenance within larval energy budgets - were in general additive, with temperature having a consistently larger effect on aerobic metabolism.

High pCO_2_ in the marine environment likely increases the maintenance costs of acid-base homeostasis, the intracellular ion balance required for protein folding and pH-sensitive physiological processes. Near-equivalent oxygen consumption rates under different pCO_2_ conditions suggest that peak-release larvae may be able to use existing pools of ion pumps to conserve acid-base homeostasis under acute stress [51], [90], [91]. However, late-release larvae may not have the capacity to buffer against the physiological demands of a simultaneously warm and acidic environment, minimizing energy requirements as a result to reduce rates of ATP synthesis. Low metabolic rates in response to OA and hypercapnia have been recorded for several taxa including sipunculid worms [92], adult and juvenile bivalves [93], jumbo squid [94], tropical fish [95], and brooded and non-brooded coral larvae [59], [60]. Other groups [96]–[98] appear to be resilient to elevated pCO_2_ conditions. Overall, metabolic depression in response to anthropogenic ocean change could have severe consequences for these late-release larvae; if it affects their abilities to navigate the water column, then larval dispersal, settlement success, and fitness may be impaired.

While larval-specific oxygen consumption values recorded here fell within the range published for *P. damicornis* larvae (*e.g.* [46], [48], [62], [63], [77]), other studies on *P. damicornis* larvae have reported different results for responses to elevated pCO_2_ and temperature levels. In March 2010 and 2012 with a population of *P. damicornis* in Taiwan, Cumbo *et al.* [48] found that for larvae released on four consecutive days, day × temperature and day x pCO_2_ x temperature had significant effects on respiration per larva. The main effect of CO_2_ on oxygen consumption was non-significant to negative, while elevated temperature stimulated aerobic metabolism. In contrast, a study with this same population conducted at the same time found no effect of pCO_2_ and a negative effect of temperature on larval respiration [63]. Following longer exposure times (1–5 days), Cumbo *et al.* [62] found that temperature, but not pCO_2_ affected mass-specific oxygen consumption in *P. damicornis* larvae from Taiwan. Interestingly, in that study, decreased oxygen consumption rates were observed in HTHC treatments for larvae at 5 days of incubation but not before. These studies differ from ours in terms of pCO_2_ exposures used, length of exposures, and biogeographic locations of coral collection (Table 6). Additionally, these studies did not document in high frequency the current environmental conditions at the collection site of their study organisms, so the interpretation of their results without knowledge of the relationship between experimental and environmental conditions is challenging. Consequently, direct comparisons of the datasets are difficult. Considering the studies together, however, highlights important differences between the larvae used here from corals in Moorea (which appear to be more sensitive to pCO_2_) and those from a genetically distinct Taiwan population [64] that experiences significantly different seawater carbonate chemistry (EB Rivest, unpublished data).

**Table 6:** Summary of studies investigating the effects of temperature and pCO_2_ on larval rates of oxygen consumption for *P. damicornis* larvae.

Study	Location	Species, Reproductive mode	Day of release	Treatment conditions	Length of exposure	O_2_ consumption (nmol O_2_ larva^−1^ min^−1^)	Effect of pCO_2_	Effect of T	Effect of Day	Interaction(s)
Cumbo *et al.* (2013) [48]	Taiwan	*Pocillopora damicornis*, brooder	Near peak-release(4 days)	25, 29°C;400, 750 µatm pCO_2_	1 d	0.068–0.262	Mostly NS, but few -	Mostly**+**	Yes	T x Day,CO_2_ x T x Day
Cumbo *et al.* (2013) [62]	Taiwan	*P. damicornis*, brooder	Peak-release only (1 day)	24, 31°C;488, 851 µatm pCO_2_^†^	1–5 d	0.077–0.188^†^	NS	**+**	N/A	T x Incubation time
Putnam *et al.* (2013) [63]	Taiwan	*P. damicornis*, brooder	Near peak-release (1 day)	24, 29°C415, 635 µatm pCO_2_	9 d	0.035–0.129	NS	–	N/A	NS
This study	Moorea, French Polynesia	*P. damicornis*, brooder	Peak-release and after(3 days)	28, 30°C450, 950 µatm pCO_2_	0.25 d	0.083–0.139	-	**+**	Yes	NS
Nakamura *et al.* (2011) [60]	Sesoko Island, Okinawa Island, Japan	*Acropora digitifera*, BS	N/A	26°C; 350, 1400, 2500 µatm pCO_2_	3, 7 d	0.002–0.005	NS, -	N/A	N/A	N/A
Albright and Langdon (2011) [59]	Summerland Key, Florida, USA	*Porites astreoides*, BS	N/A	26°C; 380, 560, 800 µatm pCO_2_	1–2 d	0.015–0.033	−	N/A	N/A	N/A

This table highlights comparisons of study location, study species (BS = broadcast spawner), release days of larvae used, duration of incubations (d = days), treatment conditions, metabolic rates, and presence/directionality of main effects and interactions (NS = not significant). ^†^Units have been converted to match other studies.

While larval-specific metabolism given as a rate per individual imparts ecological function, normalization to protein accounts for variation in mass between larvae. The effect of pCO_2_ disappeared when oxygen consumption rates were standardized by total protein content (Fig. 2B). Lower protein content of larvae incubated at High-pCO_2_, due to slower growth or down-regulation of thermotolerance pathways, could have removed the larval-specific effect of pCO_2_. Other factors could generate these respiration rates: differences in the composition of equal-sized protein pools between treatments, variation in volume of the larvae with respect to the size of the protein pool, differences in holobiont cell number, and differences in *Symbiodinium* density.

In this study, CS, a rate-limiting enzyme in the Krebs cycle, was used as a biochemical indicator for changes in metabolic function, notably mitochondrial density [87]. This methodological approach is commonly used to assess the impacts of environmental parameters and ontogeny on the metabolism of marine organisms (*e.g.* [99]–[105]); we used this enzyme assay to examine the effects of OA and temperature on the oxidative capacity of *P. damicornis* larvae. While O_2_ consumption was measured for the entire larva, oxidative capacity was measured for the animal compartment only.

Temperature and pCO_2_ did not dramatically affect larval- and protein-specific CS activity, though CS activity became suppressed at high temperature across days, particularly for late-release larvae (Fig. 3A,B). These data contrast those for O_2_ consumption, which generally show elevated rates of aerobic metabolism at higher temperatures and depressed rates at high pCO_2_. While the O_2_ consumption measurements represent the average respiration rate across the 6-hour exposure period, CS activity was a snapshot of oxidative capacity at the end of the incubation (t = 6 hours). Our O_2_ consumption measurements likely captured the peak in respiration rates while larvae were actively swimming but averaged out any variation due to developmental changes. Though all individuals used remained in the larval stage throughout the exposure, high temperatures do decrease time to metamorphosis in brooded larvae (*e.g.* [50]). As a result, minute developmental and behavioral changes as larvae progressed towards non-motile states could have generated the lower rates of CS activity found in larvae incubated at 30.6°C [36], [105], [106]. However, our interpretation is limited because the oxidative capacity of *Symbiodinium* within the larvae was not assessed. High densities of mitochondria in the *Symbiodinium* fraction at 30.6°C could have caused the effect differences between O_2_ consumption and CS activities. CS activities measured here were lower than those published for adult coral (0.0007–0.041 units mg protein^−1^ [99], [101]), whose stage-specific processes like calcification may demand higher respiration rates. Lower densities of mitochondria during larval development as well as lower surface area to volume ratios may contribute to the differences between life history stages (*e.g.* [102], [107], [108]).

An excellent proxy for the short-term metabolic responses of coral larvae, CS activity correlates well with oxidative capacity required to satisfy routine and maximal energy demands (*e.g.* [87], [109]). As measured by CS activity, larval oxidative capacity did not increase in response to elevated temperature or pCO_2_. Thus, increased demands for respiration during the short experimental exposures were likely met by an increase in the energy production of the existing pool of mitochondria through metabolic regulation of enzymes rather than *de novo* synthesis of new enzymes. Furthermore, the lack of increase in oxidative capacity at elevated temperatures despite greater flux of aerobic machinery suggests that *P. damicornis* larvae may be unable to tolerate additional stresses like OA on their energy budget. Energy demands to maintain homeostasis under higher pCO_2_ (>1000 µatm) may approach or surpass the ceiling of oxidative capacity, triggering metabolic suppression. However, following longer exposures (days to weeks), the sustained increase in energy demand imposed by acidity and warming may elicit mitochondrial biosynthesis as a compensatory response.

In this study, Q_10_ values for larval-specific rates of O_2_ consumption generally fell within the common range for chemical reaction rates (2–3; *e.g.* [81]). Notably, aerobic respiration in other brooded coral larvae (*Porites astreoides*) had Q_10_ values close to 2 [50]. Indicated by Q_10_<2, protein-specific metabolic rates were less sensitive to changes in temperature for larvae released on Day 9 and 10. For larvae released on Day 11, protein-specific O_2_ consumption rates were hypersensitive to temperature change, as shown by Q_10_>3. Oxidative capacity (*i.e.* maximum activity of CS) on all days was temperature-independent or had negative temperature dependence, with Q_10_<2 and often 1. These low Q_10_ values indicate immediate temperature compensation; many poikilotherms exhibit compensation to acute fluctuations in temperature over a portion of their natural environmental temperature range [110]. The Q_10_ patterns shown here indicate that specific components of the metabolic machinery are less sensitive to changes in temperature, but this toxicity or compensation is not detectable at the whole-organism level following acute exposures.

Rates of O_2_ consumption and CS activity of *P. damicornis* larvae are affected by pCO_2_, but not consistently, as shown by the change in sign of ΔQ_10_ between days (Table 4). The absence of dramatic changes in Q_10_ values as pCO_2_ increased indicates resistance or compensation to OA that is not revealed by biological responses of O_2_ consumption and CS activity. In general, larvae seem to be able to preserve a homeostatic level of energy metabolism under the treatment conditions, though not through the metrics that we quantified. Still, high CO_2_ levels may reduce performance of coral larvae, especially at the edges of their thermal envelope where larvae may be spending more of their energy metabolism on maintenance rather than growth or development [111]. Variable compressions or shifts in thermal tolerance windows of *P. damicornis* larvae between days could explain the Q_10_ values calculated [111]. Elevated pCO_2_ could compress the thermal envelope, resulting in lower maximum reaction rates and a narrower range of functional capacity. Alternatively, elevated pCO_2_ could shift the thermal envelope to a lower range of temperatures, decreasing the temperature for optimum functional performance.

### Variation in Physiological Plasticity among Larval Cohorts

Our results indicate that there are differences in larval sensitivity to environmental change between three cohorts that were released from adult colonies at different times. This work, along with similar studies, reveals that larvae released on different days throughout the spawning period respond differently to changes in seawater temperature and acidity (*e.g.* [49]). Larvae of *P. damicornis* and other brooding corals are known to differ by size, *Symbiodinium* density, and photophysiology throughout the spawning period ([47], [49], [50], [112]; EB Rivest, unpublished data). As larvae are stacked within coral polyps during pre-release development, variation in microenvironment by tissue depth may promote these physiological differences [113], [114]. Depending on development time within the maternal coral polyp, larvae may have different endowments of maternal *Symbiodinium* and lipid that consequently affect physiological performance. Variation in larval traits could also be a function of genotype. Due to low release numbers, genotype ratios within the larval pool were not consistent between days; however, our observations still reflect variation in biological response at the population level. Additionally, differences in larval physiology could be related with days in captivity, which was not possible to distinguish from day of release in this experiment. Variation in traits that affect physiological performance may be a product of natural selection – the range of phenotypes represented within larvae released monthly could confer selective advantage under different environmental conditions. Some phenotypes may favor retention of larvae on the natal reef, with physiology optimized for local conditions. Other phenotypes may favor longer dispersals through open-ocean ecosystems with different abiotic pH and temperature regimes.

### Natural Variation of Environmental pH and Temperature

In order to elucidate the range of environmental conditions of the water mass bathing the fringing reef where *P. damicornis* adults were collected, we measured the variability of pH and temperature on the natal coral reef in Moorea. These data were recorded as close to the site where the adult coral colonies were collected as was possible. The conditions experienced by the adult coral collected within 100 m of the sensor location may have varied (*e.g.* [115]), but our environmental data likely represent the conditions experienced by freshly-released larvae in the plankton. In February-March 2011, the average pCO_2_ on the study site (374 µatm) was similar to the global atmospheric average for the year (390 µatm, Conway and Tans, NOAA/ESRL [www.esrl.noaa.gov/gmd/ccgg/trends]). Additionally, current oscillations of pCO_2_ at this site are within projected open-ocean averages for the middle of this century [70]. The pH and temperature time series recorded during this study confirm that the average values for our experimental treatments of pCO_2_ and temperature were approximations of present-day reef conditions as well as extremes not yet observed within the seawater surrounding the fringing reef at the study site.

A key observation within the data was a 24-hour oscillation of pH (Fig. 4C,D). Larvae released from sunset to sunrise experience the nightly decrease in pH during the first part of their larval duration. The timing of the pH oscillation supports a hypothesis that within 1m of the fringing reef at this location, biological processes (respiration and photosynthesis) are driving the 24-hour pattern in the surrounding seawater. This oscillation may have been larger at the 1–2m collection depth of the adult corals used in this study. The slopes of the daily ascent and descent of pH are slightly different in absolute value and do not reflect a simple turning on and off of the balance between photosynthesis and respiration with the photoperiod. Particularly, the onset of the photosynthesis signal is delayed 5 hours after sunrise, perhaps due to calm weather reducing mixing at this time of day or lower physiological rates at the pH minima.

While fluctuations of temperature and pH are common in coral reefs and other habitats (*e.g.* [16]), variability in environmental carbonate chemistry recorded at the Moorea site differs slightly from conditions at other coral reefs [17], [116]–[118]. Compared with SeaFET pH time series data from protected reef terraces in the Northern Line islands [17], pH on the Moorea fringing reef had similar amplitudes but a higher mean value (8.075 vs. 7.958–7.981). Oceanographic features, seawater retention times, and differences in community composition may be responsible for these differences. These and other studies reporting the variability of nearshore pH and *in situ* biological responses are becoming more common (*e.g.* [17], [116], [119]–[124]) and are refining our understanding of natural variability of pH and carbonate chemistry which has historically come from open-ocean measurements (*e.g.* WOCE, http://woce.nodc.noaa.gov/wdiu; BATS, http://www.bios.edu/research/bats.html; HOTS, http://hahana.soest.hawaii.edu/hot/hot_jgofs.html).

### Implications for the Future of Coral Reefs

The over-arching outcome of this study suggests that only a portion of larvae produced monthly exhibited physiological phenotypes suited for tolerating these levels of high temperature and low pH. Furthermore, biochemical limits for increasing oxidative capacity to satisfy elevated energy demands in a warming, acidifying ocean may ultimately override the advantages offered by current phenotypes, barring acclimation and/or adaptation. If larvae cannot tolerate elevated temperatures and pCO_2_ levels by upregulating mitochondria biosynthesis to fuel stress response pathways, their demands for ATP synthesis may soon surpass the capacity of their aerobic machinery. These measured acute responses can inform a bigger picture: given longer exposures to ocean warming, acidity, and other concurrent stressors, even over multiple generations, do corals have the potential to acclimate to changing carbonate chemistries, thereby avoiding a narrowing of their thermal tolerance windows? Population-specific functional traits, such as the ones quantified here, can predict shifts in species’ ranges and phenologies in response to global climate change [125].

Comparisons between coral populations on reefs with different carbonate chemistry conditions (*e.g.* Moorea, French Polynesia and Nanwan Bay, Taiwan) may provide clues as to how physiological plasticity can be shaped by environmental variability and whether local adaptation to temperature and pH regimes buffers sensitivities to OA and rising temperature. Local adaptation in coral dinoflagellate endosymbionts has already been documented, and thermotolerant *Symbiodinium* groups may be able to enhance the tolerance and fitness of their coral host [12], [126]. Furthermore, on a global scale, there is a high degree of spatial variability in the intensity of multiple stressors for coral reefs [127], [128]. Acclimatization or local adaptation along this gradient of stress may maintain populations with suitable phenotypes for future ocean conditions. Estimates of physiological plasticity as well as contextual frameworks for variability of environmental conditions present potentially robust tools for marine conservation, allowing us to predict the influence of anthropogenic stressors on larval fitness, dispersal, and recruitment success and to manage local populations within a global context.

## Supporting Information

File S1 **Optimization of number of** ***P. damicornis*** **larvae per vial for measurements of oxygen consumption rates under pCO_2_ and temperature treatments.**
(DOC)

File S2 **Optimization of reagent concentrations for quantification of citrate synthase activity in** ***P. damicornis*** **larvae incubated in pCO_2_ and temperature treatments.**
(DOC)

File S3 **Comparisons of temperature and seawater chemistry conditions in experimental aquaria and vials during the experiment.**
(DOC)

File S4 **Protein concentrations of** ***P. damicornis*** **larvae following 6-hour exposures to combinations of pCO_2_ and temperature.** Mean ± SE concentrations of total holobiont protein for larvae used to measure rates of oxygen consumption (*n* = 6) (A) and total animal protein for larvae used to measure rates of citrate synthase activity (*n* = 3) (B). Total holobiont protein was higher at Low-pCO_2_ and on Days 10 and 11. Total animal protein at 30.6°C (vs. 27.8°C) was higher on Day 10 but lower on Day 11. Refer to Tables 2 and 3 for statistical details. Symbols are offset to improve clarity: Low-pCO_2_ at 450 µatm (circles), High-pCO_2_ at 950 µatm (triangles), 27.8°C (blue), and 30.6°C (red). Total protein values were used to assess a measure of fitness and to normalize metabolic performance under combinations of control and elevated temperature and pCO_2_. Total holobiont protein varied significantly by pCO_2_ and by Day (Table 2), with highest densities in larvae released on Days 10 and 11 (vs. Day 9; Tukey’s HSD, *p*<0.0001, 0.0214, respectively). Larvae incubated at High-pCO_2_ had slightly lower densities of total protein than at Low-pCO_2_ (Tukey’s HSD; *p* = 0.0494). Total animal protein responded differently to changes in temperature depending on day of release (Table 3) and in general was lower in larvae released on Day 9. Main effects of temperature and day were also significant (Table 3). Post-hoc analysis of the significant interaction (T x Day, Table 3) revealed significant effects of temperature on Day 9 (linear contrast with Bonferroni correction; Ζ = −2.406, *p* = 0.0484) and Day 10 (Ζ = 2.319, *p* = 0.0612), but no difference between temperatures on Day 11. The directionality of the difference in animal protein content between temperature treatments changed across days.
(TIF)
