# Supplementary material for: Responses of the Metabolism of the Larvae of Pocillopora damicornis to Ocean Acidification and Warming
Source: PLoS One. 2014 Apr 25;9(4):e96172. doi: 10.1371/journal.pone.0096172 (PMC4000220; doi:10.1371/journal.pone.0096172)
Supplement: File S1 — Optimization of number of P. damicornis larvae per vial for measurements of oxygen consumption rates under pCO2 and temperature treatments. (DOC) [file pone.0096172.s001.doc]

**S1. Optimization of number of *P. damicornis* larvae per vial for measurements of oxygen consumption rates under pCO_2_ and temperature treatments.**

The number of larvae per vial was optimized to reduce the standard deviation in the oxygen consumption per larva between replicates and to ensure that the concentration of O_2_ dropped a detectable amount over the incubation period without becoming limiting for the larvae (data not shown here). A preliminary test was conducted to determine whether O_2_ exchange differed through two vial-capping methods: gas-permeable parafilm and gas-impermeable plastic screw caps. The effect of pCO_2_ and temperature on this gas exchange was also tested. The amount of O_2_ exchange through the parafilm on vials vs. a plastic screw cap was affected only by pCO_2_ level (two-way ANOVA, F_2,99_ = 1.0574, *p* = 0.0034). Parafilm, not plastic screw caps, were used to cap vials in this experiment. However, blank vials were used in all treatments to account for background respiration, so this oxygen exchange did not contaminate the biological signal in each treatment. Exchange of CO_2_ through parafilm was not estimated; any exchange occurring would serve to equilibrate the carbonate chemistry conditions between the vial and the treatment aquarium, both of which had the same pCO_2_ treatment.
