# Supplementary material for: Responses of the Metabolism of the Larvae of Pocillopora damicornis to Ocean Acidification and Warming
Source: PLoS One. 2014 Apr 25;9(4):e96172. doi: 10.1371/journal.pone.0096172 (PMC4000220; doi:10.1371/journal.pone.0096172)
Supplement: File S2 — Optimization of reagent concentrations for quantification of citrate synthase activity in P. damicornis larvae incubated in pCO2 and temperature treatments. (DOC) [file pone.0096172.s002.doc]

**S2. Optimization of reagent concentrations for quantification of citrate synthase activity in *P. damicornis* larvae incubated in pCO_2_ and temperature treatments.**

In preliminary tests, the metabolic machinery within *Symbiodinium* in the larvae consumed reaction substrates, likely oxaloacetate, causing absorbance at 412 nm to decrease. Thus, *Symbiodinium* cells were separated from homogenates prior to initiation of the CS reaction. CS activity was tested across a range of pH of the 50 mM histidine buffer (7.0 - 8.6 at 28.0°C, *n* = 7); pH had a significant effect on CS activity (one-way ANOVA; F_6,12_ = 4.0106; *p* = 0.0151). pH 7.8 was chosen for this assay based on pairwise Student’s t tests and an *a priori* knowledge of the low intracellular pH of coral cells (*e.g.* Kühl *et al.* 1995; Venn *et al.* 2009). Substrate concentrations of acetyl-coenzyme A (0.3-0.6 mM, *n* = 7*)*, DTNB (0.1-0.35 mM, *n* = 6), and oxaloacetate (0.35-0.65 mM, *n* = 7) were also optimized and verified to be non-limiting. Results presented here for CS activity in larvae were from measurements made at 28.0 ± 0.1°C, the control temperature for culturing and respiration.

Kuhl M, Cohen Y, Dalsgaard T, Jorgensen BB, Revsbech NP (1995) Microenvironment and photosynthesis of zooxanthellae in scleractinian corals studied with microsensors for O_2_, pH, and light. Mar Ecol Progr Ser 117: 159-172.

Venn AA, Tambutté E, Lotto S, Zoccola D, Allemand D, Tambutté S (2009) Imaging intracellular pH in a reef coral and symbiotic anemone. P Natl A Sci USA 106: 16574-16579.
