# Supplementary material for: Responses of the Metabolism of the Larvae of Pocillopora damicornis to Ocean Acidification and Warming
Source: PLoS One. 2014 Apr 25;9(4):e96172. doi: 10.1371/journal.pone.0096172 (PMC4000220; doi:10.1371/journal.pone.0096172)
Supplement: File S3 — Comparisons of temperature and seawater chemistry conditions in experimental aquaria and vials during the experiment. (DOC) [file pone.0096172.s003.doc]

**S3. Comparisons of temperature and seawater chemistry conditions in experimental aquaria and vials during the experiment.**

Temperature as well as tank pH differed between treatments (F_3,69_ = 1960.59, *p* < 0.0001; F_3,23_ = 3813.58, *p* < 0.0001). pH of seawater within the respirometry vials also varied significantly with treatment (F_3,23_ = 2663.83, *p* < 0.0001), though absolute vial pH was slightly different from tank pH due to filtration and handling while vials were filled. While pH differed between tanks and vials within each treatment (LTLC: F_1,11_ = 41.37, *p* < 0.0001; LTHC: F_1,11_ = 15.04, *p* = 0.0031; HTLC: F_1,11_ = 4.88, *p* = 0.0517; HTHC: F_1,11_ = 27.31, *p* = 0.0004), the pCO_2_ treatment conditions did not overlap. Salinity was significantly higher in the HTHC treatment (F_3,11_ = 6.67, *p* = 0.0144), while A_T_ did not vary significantly between temperature and pCO_2_ combinations. Similarly, pCO_2_ was significantly different between treatments in tanks (F_3,11_ = 1247.34, *p* < 0.0001) and in vials (F_3,11_ = 651.93, *p* < 0.0001). pCO_2_ levels of tanks and vials within treatments were different (LTLC: F_1,5_ = 11.32, *p* = 0.0282; LTHC: F_1,5_ = 2.30, *p* = 0.2044; HTLC: F_1,5_ = 2.53, *p* = 0.1868; HTHC: F_1,5_ = 14.93, *p* = 0.0181), but both tanks and vials grouped by treatment (Low- or High-pCO_2_).
