# Supplementary figures and images for: Responses of the Metabolism of the Larvae of Pocillopora damicornis to Ocean Acidification and Warming
Source: PLoS One. 2014 Apr 25;9(4):e96172. doi: 10.1371/journal.pone.0096172 (PMC4000220; doi:10.1371/journal.pone.0096172)

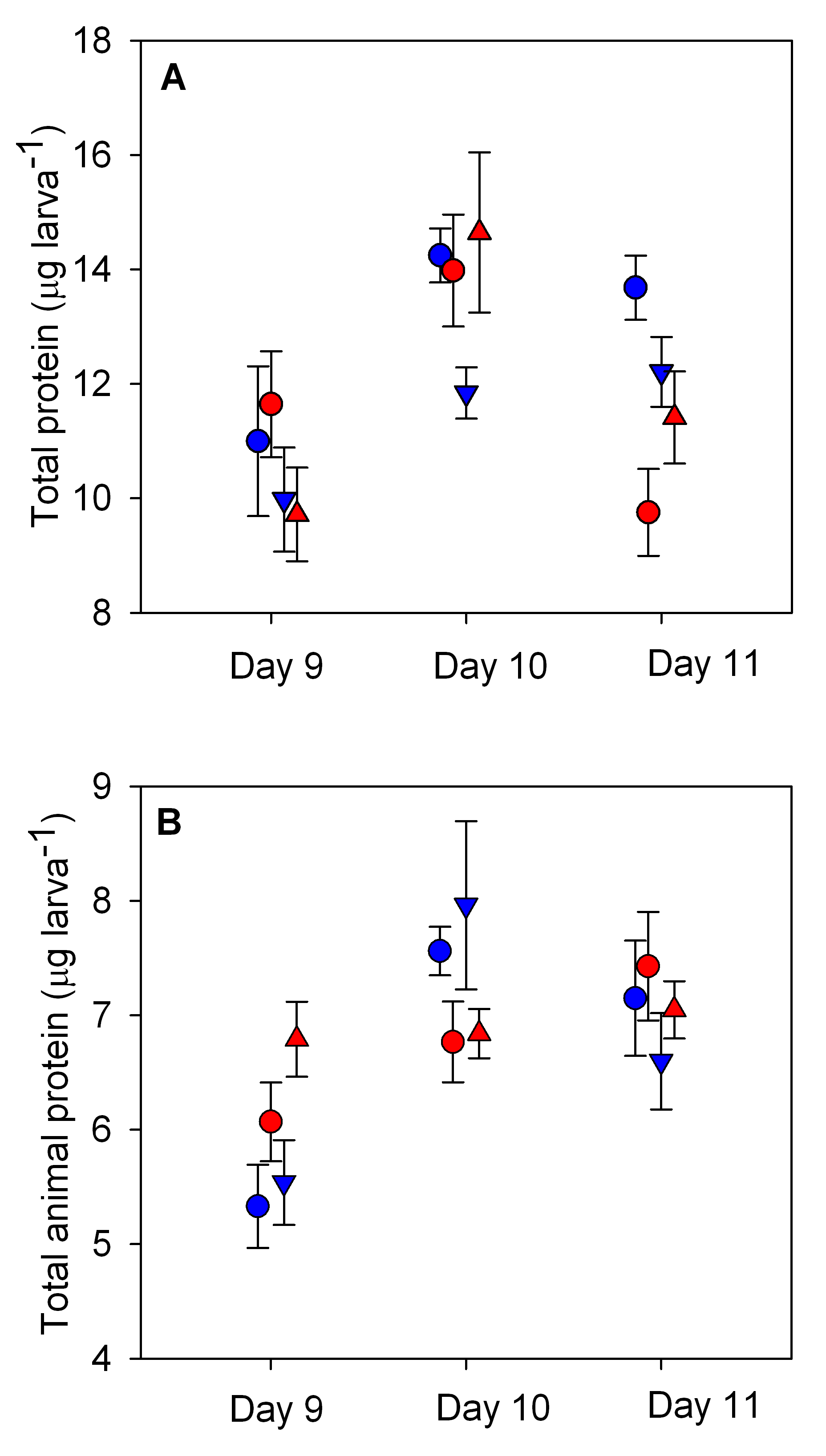

Supplement: File S4 — Protein concentrations of P. damicornis larvae following 6-hour exposures to combinations of pCO2 and temperature. Mean ± SE concentrations of total holobiont protein for larvae used to measure rates of oxygen consumption (n = 6) (A) and total animal protein for larvae used to measure rates of citrate synthase activity (n = 3) (B). Total holobiont protein was higher at Low-pCO2 and on Days 10 and 11. Total animal protein at 30.6°C (vs. 27.8°C) was higher on Day 10 but lower on Day 11. Refer to Tables 2 and 3 for statistical details. Symbols are offset to improve clarity: Low-pCO2 at 450 µatm (circles), High-pCO2 at 950 µatm (triangles), 27.8°C (blue), and 30.6°C (red). Total protein values were used to assess a measure of fitness and to normalize metabolic performance under combinations of control and elevated temperature and pCO2. Total holobiont protein varied significantly by pCO2 and by Day (Table 2), with highest densities in larvae released on Days 10 and 11 (vs. Day 9; Tukey’s HSD, p<0.0001, 0.0214, respectively). Larvae incubated at High-pCO2 had slightly lower densities of total protein than at Low-pCO2 (Tukey’s HSD; p = 0.0494). Total animal protein responded differently to changes in temperature depending on day of release (Table 3) and in general was lower in larvae released on Day 9. Main effects of temperature and day were also significant (Table 3). Post-hoc analysis of the significant interaction (T x Day, Table 3) revealed significant effects of temperature on Day 9 (linear contrast with Bonferroni correction; Ζ = −2.406, p = 0.0484) and Day 10 (Ζ = 2.319, p = 0.0612), but no difference between temperatures on Day 11. The directionality of the difference in animal protein content between temperature treatments changed across days. (TIF) [file pone.0096172.s004.tif]
